# Cost-Effectiveness of Newborn Circumcision in Reducing Lifetime HIV Risk among U.S. Males

**DOI:** 10.1371/journal.pone.0008723

**Published:** 2010-01-18

**Authors:** Stephanie L. Sansom, Vimalanand S. Prabhu, Angela B. Hutchinson, Qian An, H. Irene Hall, Ram K. Shrestha, Arielle Lasry, Allan W. Taylor

**Affiliations:** Division of HIV/AIDS Prevention, National Center for HIV/AIDS, Viral Hepatitis, STD, and TB Prevention, Centers for Disease Control and Prevention, Atlanta, Georgia, United States of America; Tulane University, United States of America

## Abstract

**Background:**

HIV incidence was substantially lower among circumcised versus uncircumcised heterosexual African men in three clinical trials. Based on those findings, we modeled the potential effect of newborn male circumcision on a U.S. male's lifetime risk of HIV, including associated costs and quality-adjusted life-years saved.

**Methodology/Principal Findings:**

Given published estimates of U.S. males' lifetime HIV risk, we calculated the fraction of lifetime risk attributable to heterosexual behavior from 2005–2006 HIV surveillance data. We assumed 60% efficacy of circumcision in reducing heterosexually-acquired HIV over a lifetime, and varied efficacy in sensitivity analyses. We calculated differences in lifetime HIV risk, expected HIV treatment costs and quality-adjusted life years (QALYs) among circumcised versus uncircumcised males. The main outcome measure was cost per HIV-related QALY saved. Circumcision reduced the lifetime HIV risk among all males by 15.7% in the base case analysis, ranging from 7.9% for white males to 20.9% for black males. Newborn circumcision was a cost-saving HIV prevention intervention for all, black and Hispanic males. The net cost of newborn circumcision per QALY saved was $87,792 for white males. Results were most sensitive to the discount rate, and circumcision efficacy and cost.

**Conclusions/Significance:**

Newborn circumcision resulted in lower expected HIV-related treatment costs and a slight increase in QALYs. It reduced the 1.87% lifetime risk of HIV among all males by about 16%. The effect varied substantially by race and ethnicity. Racial and ethnic groups who could benefit the most from circumcision may have least access to it due to insurance coverage and state Medicaid policies, and these financial barriers should be addressed. More data on the long-term protective effect of circumcision on heterosexual males as well as on its efficacy in preventing HIV among MSM would be useful.

## Introduction

Three randomized, controlled clinical trials conducted in South Africa, Kenya, and Uganda found that medical circumcision in men reduced participants' risk of HIV infection [1]–[3]. In these studies, men who had been randomly assigned to the circumcision group had a lower (60% in South Africa, 53% in Kenya, and 51% in Uganda) incidence of HIV infection compared with men assigned to the wait list group to be circumcised at the end of the study. In a per protocol analysis, men who had been circumcised had a 76% (South Africa), 60% (Kenya), and 55% (Uganda) reduction in risk of HIV infection compared to those who were not circumcised. In Kenya, efficacy increased to 64% when the follow-up period was extended from 24 to 42 months (Bailey RC, Moses S, Parker CB, Agot K, Maclean I, et al. The protective effect of male circumcision is sustained for at least 42 months: results from the Kisumu, Kenya trial. *XVII International AIDS Conference*. Aug. 3–8, 2008. Mexico City, Mexico.).

Based on the results of these studies, the World Health Organization (WHO) has recommended that male circumcision be recognized as an efficacious intervention for HIV prevention in countries and regions with heterosexual epidemics, high HIV and low male circumcision prevalence. Circumcision should be considered as part of a comprehensive HIV prevention package and not a replacement for known methods of HIV prevention [4]. The WHO noted that the partially-protective effect of male circumcision for heterosexual men was remarkably consistent across observational studies as well as across the three randomized, controlled clinical trials assessed in this paper [5].

In the United States, there are limited observational data on the protective efficacy of circumcision for heterosexual males; and the methods used in existing studies differ. A 2008 cross-sectional study of African-American males attending a sexually transmitted disease clinic, and with known exposure to HIV, indicated an adjusted HIV prevalence rate ratio of 0.49 (95% Confidence Interval [CI]: 0.26–0.93) among circumcised men compared with uncircumcised men [6]. A 1993 prospective study of men attending an inner-city sexually transmitted disease clinic, whose exposure to HIV was calculated based on participants' reports of sex with women of various risk groups, found uncircumcised men had an adjusted odds ratio of HIV seroconversion of 3.5. The 95% confidence interval, however, was 0.8 to 15.8 [7].

Among men who have sex with men (MSM), results from a meta-analysis of 15 observational studies on male circumcision did not find a statistically significant association between circumcision and HIV status [8]. An analysis of Australian MSM with a preference for the insertive role in anal intercourse found a significant reduction in HIV incidence among circumcised men with HIV-infected partners or those whose HIV status was unknown, compared with uncircumcised men (hazard rate 0.11, 95% CI: 0.03–0.8, p = 0.041) [9].

Although mathematical models have demonstrated a potentially large reduction in HIV incidence among heterosexuals in Africa as the practice of circumcision increases [10]–[13], the potential impact of circumcision is less well-understood in the United States, where the majority of HIV infection among U.S. males occurs through sexual contact with other males, and the prevalence of male circumcision already is high [14], [15]. We examine the potential effectiveness and cost-effectiveness of newborn circumcision on reducing a U.S. male's lifetime risk of HIV by applying efficacy data from the African trials to the portion of U.S. males' lifetime HIV risk related to heterosexual contact.

## Methods

Institutional review board consideration was not required for this work because it did not involve the collection or analysis of primary data. We developed a static Excel-based (Version 2003, Microsoft Corporation, WA) decision model to compare the HIV-related costs and quality-adjusted life-years among U.S. males who are circumcised at birth with those who are not. We based our analysis on previously published estimates of lifetime HIV risk for U.S. males[16]. We calculated the expected difference in lifetime HIV risk among circumcised and uncircumcised males given the portion of lifetime HIV risk attributed to heterosexual contact, the prevalence of circumcision in the United States and the observed efficacy of circumcision on HIV prevention in Africa. Our work is consistent with reference-case recommendations of the Panel on Cost-Effectiveness in Health and Medicine for conducting and reporting cost-effectiveness analyses, including the use of a societal perspective [17].

Analytic parameters from published sources are in Table 1. Lifetime HIV risk for U.S. males overall, and by race/ethnicity, has been estimated at 1.87%, 0.96%, 2.88% and 6.23% among all, white, Hispanic and black males, respectively[16].

**Table 1:** Key parameters for assessing the cost-effectiveness of newborn circumcision in reducing lifetime HIV risk among U.S. males.

Variable (reference)	Base	Range
Lifetime HIV risk among U.S. males[16] (%) All Black Hispanic White	1.87 6.23 2.88 0.96	95% CI* 1.86–1.89 6.14–6.33 2.78–2.99 0.95–0.98
Lifetime circumcision efficacy (%) Heterosexually acquired HIV[1]–[3] HIV acquired through sex with men†	60 0	95% CI 39–80 0–20
Prevalence of male circumcision[15] (%) All Black Hispanic White	79 73 42 88	95% CI 77–80 69–77 40–45 87–90
Average age of HIV infection among men (years)‡	34	32–36
Remaining life expectancy after HIV infection (years) [23]	32	32
Total life expectancy without HIV infection (years) [24]	77	77
Expected quality-adjusted life-years without HIV infection, discounted to birth	30.81	30.37–30.91
Expected quality-adjusted life-years with HIV infection, depending on year of infection, discounted to birth [31]	28.66	27.94–28.82
Lifetime cost of HIV discounted to birth ($) [23]	127,298	81,109–165,487
Cost of neonatal circumcision ($) [25], [29]	257	216–601
Discount rate (%)	3.0	1.0–5.0

* CI = Confidence Interval.

†Assumption.

‡Written communication from R Song, Division of HIV/AIDS Prevention, Centers for Disease Control and Prevention, August 2008.

Based on the African adult circumcision trial data, we assumed the efficacy of circumcision for the prevention of heterosexually acquired HIV was 60% over a lifetime, compared with uncircumcised men. In sensitivity analyses, we averaged the lower and upper bounds from the 95% confidence intervals reported in the per-protocol analyses from the African trials, 39% and 80% [1]–[3]. We assumed in the base case that circumcision conferred no protection for the MSM transmission category. In a sensitivity analysis, we assumed as high as a 20% efficacy for that category [8], [9], [18]–[21].

The prevalence of circumcision among U.S. males born from 1940 through 1979 was 79% overall, 88% for white males, 73% for black males and 42% for Mexican American males [15]. We applied the circumcision prevalence for Mexican Americans to all Hispanic males. In sensitivity analyses we used the lower and upper limits of the 95% confidence interval for circumcision prevalence among all males and specific to race and ethnicity.

We chose 34 years as the median age of HIV infection among U.S. males based on 2006 HIV incidence data (written communication from R Song, Division of HIV/AIDS Prevention, Centers for Disease Control and Prevention, August 2008. See also [22]). In a sensitivity analysis, we varied the median age of infection between 30 and 38 years. We assumed a life expectancy of 32 additional years for males infected with HIV, and HIV lifetime treatment costs discounted to the time of infection of $343,129 [23]. We further discounted HIV lifetime treatment costs at 3% per year from the age of infection to birth to generate lifetime HIV treatment costs of $127,298. In a sensitivity analysis, we varied lifetime HIV treatment costs by 25%. We also varied the discount rates for lifetime treatment costs and quality-adjusted life-years from 0% to 5%. We assumed that uninfected men had an average life expectancy of 77 years [24].

The cost of newborn circumcision often includes that for the physician who performs the procedure and facility-related costs. We estimated a cost of $257 and a range of $216 to $601, based on 4 published cost estimates plus a review of newborn circumcision costs in the MarketScan Medicaid database [25]–[29]. All costs in the study are adjusted to $US2007, using the medical care component of the Consumer Price Index.

For all males and by race/ethnicity, we calculated the fraction of lifetime HIV risk attributable to each transmission category. The calculation was based on the transmission categories associated with 2005–2006 new HIV diagnoses of males in the HIV/AIDS Reporting System in 33 name-based reporting states (written communication, M. Campsmith, Division of HIV/AIDS Prevention, Centers for Disease Control and Prevention, July 2008. See also [14], [30]). We used the following transmission categories: MSM, high-risk heterosexuals (HRH), intravenous drug users (IDU), and others. The HIV/AIDS data also contain the transmission category MSM/IDU. To simplify our calculations, we assigned the males in the MSM/IDU HIV transmission category to either the MSM-only or IDU-only transmission categories. The assignment of those in the MSM/IDU transmission category was proportionate to the number of males in the MSM-only compared with IDU-only category.

The majority of HIV diagnoses (71.4%) for all U.S. males were associated with sex with men, a route of transmission for which the efficacy of circumcision appears to be quite limited (Table 2). The proportion of HIV infections attributable to heterosexual contact was 15.7% for all males, with considerable variation across race/ethnicity, ranging from 6.7% for white males to 23.1% for black males. In sensitivity analyses, we varied the fraction of lifetime risk attributable to heterosexual contact by 25%.

**Table 2:** U.S. males' HIV lifetime risk by transmission category.

	All males		Black		Hispanic		White	
Lifetime HIV Risk (%) 95% CI	1.87 1.86–1.89		6.23 6.14–6.33		2.88 2.78–2.99		0.96 0.95–0.98	
Transmission category	Attributable % of HIV diagnoses	Attributable portion of lifetime HIV risk	Attributable % of HIV diagnoses	Attributable portion of lifetime HIV risk	Attributable % of HIV diagnoses	Attributable portion of lifetime HIV risk	Attributable % of HIV diagnoses	Attributable portion of lifetime HIV risk
MSM	71.4	1.33	61.5	3.83	69.2	1.99	84.5	0.81
HRH	15.7	0.29	23.1	1.44	16.2	0.47	6.7	0.06
IDU	12.5	0.23	14.9	.93	14.2	0.41	8.5	0.08
Other	0.4	0.01	<.01	0.02	0.4	0.01	0.4	<0.01

In our base case analysis, we assumed that the calculated fraction of lifetime HIV risk attributable to heterosexual activity reflected a weighted average of the risk among circumcised and uncircumcised males, and that the lifetime HIV risk of circumcised males was 60% lower than the lifetime HIV risk of uncircumcised males. We calculated the lifetime risk of HIV attributable to heterosexual activity separately for circumcised and uncircumcised males as follows:
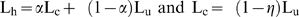



Where:
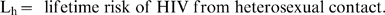


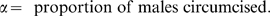


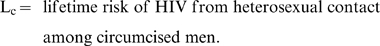


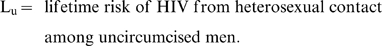


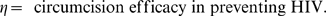



We then summed the fractions of lifetime risk of HIV associated with each transmission category separately for circumcised and uncircumcised men to estimate total lifetime HIV risk. In the base case, we assumed the lifetime risk of HIV associated with sex with men, intravenous drug users, and other transmission categories was the same for uncircumcised men as for circumcised men; only lifetime risk associated with sex with women was different between the groups of men. We calculated the percentage of difference in lifetime risk for circumcised versus uncircumcised men and the number of circumcisions needed to prevent one HIV infection.

We estimated expected lifetime HIV costs among circumcised and uncircumcised males by multiplying each group's lifetime risk of HIV by HIV treatment costs discounted to the time of birth. We derived expected savings in treatment costs among circumcised men by subtracting expected lifetime HIV treatment costs among those men from expected lifetime HIV treatment costs among uncircumcised men. We estimated net circumcision costs among circumcised males by subtracting the expected savings in HIV treatment dollars from the cost of neonatal circumcision. We used published HIV-related utility estimates to estimate the expected the number of QALYs saved among circumcised men compared with uncircumcised males [31].

We performed one-way and multi-way sensitivity analyses to estimate the effect of various values for each parameter on incremental cost-effectiveness ratios. We conducted probabilistic sensitivity analyses, simulating 10,000 iterations for all males and for black, Hispanic, and white males, to determine the proportion of incremental cost-effectiveness ratios that fell within thresholds ranging from cost-saving to $200,000 per QALY saved [32]–[34].

## Results

We estimated that at 60% lifetime efficacy, circumcision would reduce the lifetime risk of HIV diagnosis for all males by 15.7% in the base case analysis, and the reduction ranged from 7.9% among white males to 20.9% for black males (Table 3). The number of circumcisions needed to prevent one HIV infection was 298 for all males, and ranged from 65 for black males to 1,231 for white males. For all males, circumcision increased undiscounted HIV-related quality-adjusted life expectancy by 19.6 days, and discounted (at 3%) quality-adjusted life expectancy by 2.6 days. For all males, circumcision resulted in undiscounted lifetime HIV-related healthcare savings of $2,070 per male and discounted lifetime HIV-related healthcare savings of $427. Newborn circumcision resulted in savings in costs and in quality-adjusted life-years for all males and for black and Hispanic males. For white males, newborn circumcision cost $87,792 for each QALY saved, under base case assumptions.

**Table 3:** Key cost-effectiveness outcomes for newborn circumcision and the reduction of lifetime HIV risk among U.S. males.

	All males	Black	Hispanic	White
Lifetime HIV risk without circumcision (%)	2.14	7.35	3.04	1.03
Lifetime HIV risk with circumcision (%)	1.80	5.82	2.66	0.95
Reduction in lifetime HIV risk (%)	15.7	20.9	12.3	7.9
Number needed to treat to prevent 1 HIV infection	298	65	268	1,231
Expected lifetime HIV treatment costs (discounted) without circumcision ($)	2,718	9,359	3,866	1,313
Expected lifetime HIV treatment costs (discounted0 with circumcision ($)	2,291	7,402	3,391	1,210
Savings in expected lifetime HIV treatment costs ($)	427	1,956	475	103
Net provider costs ($)	−170	−1,699	−218	154
Expected QALYs (discounted) with circumcision	30.77	30.68	30.75	30.787
Expected QALYs (discounted) without circumcision	30.76	30.65	30.74	30.785
QALYs saved	.007	.033	.008	.002
Net costs/QALY saved ($)	Cost saving	Cost saving	Cost saving	87,792

The variable with the greatest impact on the incremental cost-effectiveness ratio in one-way sensitivity analyses was the discount rate, followed by circumcision efficacy in preventing heterosexually acquired HIV, circumcision cost, and circumcision efficacy in preventing HIV acquired through sex with men (Table 4). A two-way sensitivity analysis combining the effect of circumcision efficacy in preventing heterosexually acquired HIV and the cost of newborn circumcision indicated the highest incremental cost-effectiveness ratio for all males is $102,789 when efficacy was lowest (40%) and the cost of circumcision was highest ($600) (Table 5).

**Table 4:** One-way sensitivity analyses: incremental cost of newborn circumcision per quality-adjusted life-year saved for all U.S. males and by race/ethnicity.

	All males ($)	Black males ($)	Hispanic males ($)	White males ($)
Base case	CS	CS*	CS	87,792
Discount rate = 1%	CS	CS	CS	CS
Discount rate = 5%	52,778	CS	40,740	429,923
Heterosexual efficacy = 39%	12,816	CS	CS	255,422
Heterosexual efficacy = 80%	CS	CS	CS	9,964
Cost of circumcision = $216	CS	CS	CS	64,351
Cost of circumcision = $601	24,011	CS	15,697	284,474
MSM efficacy = 5%	CS	CS	CS	37,402
MSM efficacy = 10%	CS	CS	CS	11,018
MSM efficacy = 20%	CS	CS	CS	CS
Proportion of HIV risk from heterosexual contact: 25% decrease from baseline	CS	CS	CS	136,772
Proportion of HIV risk from heterosexual contact: 25% increase from baseline	CS	CS	CS	58,404
Age of HIV infection = 30 years	CS	CS	CS	60,377
Age of HIV infection = 38 years	CS	CS	CS	133,730
Discounted lifetime HIV treatment costs = $89,109	CS	CS	CS	105,536
Discounted lifetime HIV treatment costs = $165,487	CS	CS	CS	70,048
Circumcision prevalence: lower limit of 95% confidence	CS	CS	CS	89,660
Circumcision prevalence: upper limit of 95% confidence interval	CS	CS	CS	84,057

CS* = cost saving.

**Table 5:** Two-way sensitivity analysis of efficacy of circumcision related to heterosexually-transmitted HIV and cost of newborn circumcision on incremental cost per HIV-related quality-adjusted life-year saved for all U.S. males.

	Lifetime efficacy of newborn circumcision in preventing heterosexually-acquired HIV among U.S. males								
Cost of newborn circumcision	40%	45%	50%	55%	60%	65%	70%	75%	80%
$200	CS*	CS	CS	CS	CS	CS	CS	CS	CS
$250	$8,326	CS	CS	CS	CS	CS	CS	CS	CS
$300	$21,821	$8,668	CS	CS	CS	CS	CS	CS	CS
$350	$35,316	$19,971	$7,695	CS	CS	CS	CS	CS	CS
$400	$48,811	$31,274	$17,244	$5,765	CS	CS	CS	CS	CS
$450	$62,305	$42,576	$26,793	$13,879	$3,118	CS	CS	CS	CS
$500	$75,800	$53,879	$36,342	$21,993	$10,036	CS	CS	CS	CS
$550	$89,295	$65,181	$45,891	$30,107	$16,955	$5,825	CS	CS	CS
$600	$102,789	$76,484	$55,439	$38,221	$23,873	$11, 732	$1,325	CS	CS

* Cost saving.

In a probabilistic sensitivity analysis based on 10,000 iterations, newborn circumcision was cost-saving for 78.3%, 100%, 88.3% and .3% of iterations for all, black, Hispanic, and white males, when no efficacy was assigned to preventing HIV among MSM. It was cost-saving for 91.0%, 100%, 97.1% and 3.3% of iterations for the same groups when a 5% efficacy was assumed for MSM (Table 6). For white males, newborn circumcision was cost-effective for 67.3% of iterations at a threshold of $150,000 per QALY saved, and 82.4% of iterations at a threshold of $200,000.

**Table 6:** Probability of the cost of newborn circumcision per HIV-related quality-adjusted life year saved falling within selected thresholds for all, black, Hispanic and white U.S. males, with efficacy for the prevention of HIV among MSM equal to 0% and 5%.

	MSM efficacy = 0%			
Threshold cost-effectiveness values ($)	All	Black	Hispanic	White
Cost saving	78.3%	100%	88.3%	0.3%
50,000	99.3%	100%	99.9%	13.7%
100,000	100%	100%	100%	41.9%
150,000	100%	100%	100%	67.3%
200,000	100%	100%	100%	82.4%
	MSM efficacy = 5%			
Cost saving	91.0%	100%	97.1%	3.3%
50,000	100%	100%	100%	46.5%
100,000	100%	100%	100%	82.0%
150,000	100%	100%	100%	94.8%
200,000	100%	100%	100%	98.6%

## Discussion

A U.S. male has a 1.87% chance of becoming infected with HIV over his lifetime, and the risk varies substantially by race and ethnicity, from 0.94% among white males to 6.22% among black males. Using randomized clinical trial results from Africa, our analysis shows that newborn circumcision can reduce the lifetime risk of HIV, and that the protective effect also varies by race/ethnicity. The reduction is 16% for all males, nearly 21% for black males, and 8% for white males, given the base case assumption that the protective effect of circumcision applies only to heterosexually acquired HIV.

Our analysis indicates that racial and ethnic groups who would potentially benefit the most from newborn circumcision because they are at greater risk of HIV transmission through heterosexual contact, currently, have a lower prevalence of circumcision than white males. Circumcision prevalence was 73% for black males and 42% for Mexican American males, compared with 88% among white males [15]. Based on our estimated number needed to treat and the 2006 male birth cohorts for blacks (314,670), Hispanics (530,971), and whites (1,184,120), if the entire racial/ethnic cohort were circumcised instead of the proportions reported above, then 1,307 HIV infections would be prevented among the cohort of black males, or 6.7% of those expected over their lifetimes, 1,149 (7.5%) cases would be prevented among Hispanic males, and 115 (1.0%) of those expected among white males. [35]

Parents, in consultation with their physician, family members and other health care professionals, decide whether newborn circumcision is performed, and these decisions often are made based on religious or cultural grounds. The decision may be constrained, however, by health care reimbursement policies. In a 1995 review, 61% of circumcisions were paid for by private insurance, 36% by Medicaid, and 3% by the parents [36]. In 1999, the American Academy of Pediatrics revised its policy on newborn circumcision to state that “existing scientific evidence demonstrates potential medical benefits of newborn male circumcision; however, these data are not sufficient to recommend routine neonatal circumcision [37].” Currently, a number of states have eliminated Medicaid payments for circumcisions not deemed medically necessary [38]. In states whose Medicaid program covers neonatal circumcision, rates were reported to be more than twice as high (69.6%) as in states whose Medicaid program does not pay for male circumcision (31.2%) [39]. In these latter states, populations most likely to benefit from newborn circumcision may be least able to obtain it. In 2005, 40% of Hispanic children and 46% of black children were covered by Medicaid, compared with 19% of white children [40].

Our results show that newborn circumcision is usually cost saving in the United States because of the low cost of the procedure, current lifetime risk of HIV among U.S. males and the high cost of treating HIV. Previous economic evaluations of newborn circumcision in the U.S. were published before data on HIV prevention from the African trials became available. Those studies typically focused on costs and benefits of circumcision-associated conditions other than HIV and other STIs. Even when these benefits were included, the magnitude of the benefit and the lifetime HIV risk among males were not as well understood. One study found the expected lifetime cost of circumcision was small, compared with larger expected benefits [25]. Two of the studies estimated that both costs and benefits were too small to play an influential role in the decision whether to perform the procedure [27], [28]. One study found that negative outcomes associated with circumcision outweighed the benefits [26].

Newborn circumcision for white males in the United States did not generate cost-saving results. White males already have a high prevalence of circumcision (88%), a low lifetime risk of HIV (0.96%) and a low risk of acquiring HIV through heterosexual contact (6.7%) compared with black and Hispanic males, so additional circumcisions provide little benefit. The incremental cost-effectiveness ratio for the base case analysis for white males was $87,792. A probabilistic sensitivity analysis showed that the incremental cost-effectiveness ratio fell below $150,000 67.3% of the time and below $200,000 82% of the time. Historically, a common cost-effectiveness threshold in the U.S. has been $50,000 per quality-adjusted life-year saved. A more recent analysis of society's current willingness to pay for an extra year of life suggested a range of $183,000 to $264,000 [32]. Others have suggested a threshold approaching $200,000 or more [33]. The World Health Organization considers a country-specific threshold equal to three times the country's per-capita gross domestic product [34]. In 2007, the U.S. per capita gross domestic product was $46,800, or $140,400 when tripled [41].

The choice of discount rate had the biggest effect on the incremental cost-effectiveness ratio. The impact of discounting is particularly large in this analysis because the intervention costs are assumed immediately at birth, but the prevention benefits do not begin to accrue until more than three decades later. In our base case, we used the recommended 3% discount rate [17]. Further guidance on how best to discount benefits that occur in adulthood following interventions delivered in childhood would be helpful.

Other important factors in the one-way sensitivity analysis were lifetime efficacy of circumcision in preventing heterosexually-acquired HIV and the cost of newborn circumcision. However, even the least favorable inputs generated cost-effective results for all males. Nonetheless, more research on the long-term efficacy of circumcision would be useful.

We note that even a modest efficacy in preventing HIV transmission among MSM makes the procedure more cost-effective. For white males, an efficacy of 5% improves the cost-effectiveness ratio from $87,792 in the base case to $37,402. An efficacy of 20% makes the procedure cost saving for white males. Currently, there are no data from randomized clinical trials on the benefits of circumcision in preventing HIV among MSM. These data would be useful in determining the impact of newborn circumcision on HIV epidemics in developed countries where a significant number of HIV infections occur through sex among MSM.

Our analysis has two limitations that, if considered, would make neonatal circumcision more cost-effective. First, our analysis did not include other health benefits associated with the procedure. Lack of male circumcision has been associated with increased incidence of sexually transmitted ulcer disease, infant urinary tract infections, penile cancer, and cervical cancer in the female partners of uncircumcised men [42]. One meta-analysis of the association between male circumcision and risk of genital ulcer disease concluded that there was a significantly lower risk of syphilis and chancroids among circumcised men but less effect on HSV-2 [43]. Further analyses of the randomized, controlled circumcision trial in Uganda found a 28% decreased cumulative probability of HSV-2 over 24 months and a lower prevalence of high-risk HPV genotypes, but no significant difference in the incidence of syphilis among circumcised trial participants compared with those who were not circumcised [44]. Subsequent multivariate analyses of the South African trial data found a 34% decrease in the incidence of HSV-2 over 21 months among circumcised compared with uncircumcised males [45].

Second, we did not count secondary HIV transmissions that would be prevented among partners of circumcised males who remained uninfected due to circumcision. Models showing the benefits of circumcision in Africa indicate benefit to female partners over time as HIV prevalence among men declines [10]–[13].

We did not include two factors that could make neonatal circumcision for HIV prevention less cost-effective. One was the cost of adverse events associated with newborn circumcision. In large studies of newborn circumcision in the U.S., complication rates ranged from 0.2% to 2%, most commonly minor bleeding and local infection [42]. Another study found a complication rate of .22% (mostly bleeding) among newborns who were circumcised before discharge from the hospital, compared with .01% among those who were not circumcised. The circumcised newborns with complications had an average hospital stay of 2.81 days compared with 2.26 days among those circumcised but without complications [46].

Also, we did not attempt to model potential changes in risky sexual behaviors among circumcised men. The South African circumcision trial showed that men in the intervention group had significantly more sex acts (but not partners) over the course of the trial, although the protective effect of circumcision remained [1]. The Kenyan and Ugandan trials reported that circumcised men did not practice riskier sexual behaviors during those trials [2], [3]. In Kenya, risk behaviors among circumcised and uncircumcised men declined over a 12-month period during the trial [47]. As the benefits of circumcision in preventing heterosexually acquired HIV become more widely known, circumcised men and their partners may practice riskier sexual behaviors. On the other hand, men who have been circumcised since birth may be less likely to take their circumcision status into account when determining the level of risk acceptable to them and their partners. Sexual risk practices should be monitored over time through surveys and safe sex practices should continue to be encouraged among circumcised males and their partners.

We based our analysis on current estimates of lifetime HIV risk, HiV transmission categories, circumcision prevalence, and costs of both newborn circumcision and lifetime HIV treatment. These estimates could change in ways that might make neonatal circumcision more or less cost-effective over the lifetime of a male born today.

Although our study accounted for the differences U.S. and African males in HIV and circumcision prevalence and mode of HIV transmission, we assumed the protective effect of circumcision observed in the African trials was applicable to U.S. males. The efficacy of circumcision in all three of the randomized African trials, which occurred in three different countries, was remarkably similar. Randomizing participants to immediate or delayed circumcision is likely to have controlled for other factors that would have made HIV acquisition more or less likely to occur in the intervention versus the control groups. It is possible that the protective effect of circumcision in the African trials was due to the prevention of HSV-2, which then prevented the acquisition of HIV, and so the protective effect of circumcision in the United States would be reduced because of the lower HSV-2 prevalence among U.S. males. However, investigators from the South African trial reported that the protective effect of circumcision appeared to be independent of HSV-2 serostatus. Moreover, the prevalence of HSV-2 among the South African trial participants was similar to that among U.S. males [45], [48]. Investigators from the Ugandan trial reported that genital ulcer disease played at most a modest role in the protection of HIV afforded by circumcision [49]. Thus, while the absolute incidence of HIV observed among heterosexual men in the African trials is much larger than that among U.S. heterosexual males, we assumed the relative 60% decrease in heterosexual transmission among circumcised compared with uncircumcised males would hold true regardless of the underlying prevalence of HIV, circumcision or HSV-2.

This paper evaluates the efficacy of newborn circumcision solely in the prevention of HIV, and it indicates that the procedure is cost saving under most scenarios. The greatest risk reduction occurs for black and Hispanic males. Although our analysis suggests that newborn circumcision will not have a large impact on the HIV epidemic in the United States, it could play a role in reducing the number of new cases of HIV; particularly when used with other efficacious prevention interventions. Considering variations in lifetime risk of HIV and circumcision prevalence among racial and ethnic groups in the U.S., newborn circumcision may provide one additional tool in reducing longstanding disparities in HIV incidence [50]. Financial barriers that prevent parents from having the choice to circumcise their male newborns should be reduced or eliminated.
